# 113. Reliability of Nasopharyngeal PCR for the Detection of Otopathogens in Children with Uncomplicated Acute Otitis Media

**DOI:** 10.1093/ofid/ofab466.113

**Published:** 2021-12-04

**Authors:** Holly M Frost, Thresia Sebastian, Amy Keith, Melanie Kurtz, Andreas Bress, Richard Egan, Samuel R Dominguez, Samuel R Dominguez, Sarah Parker, Timothy C Jenkins

**Affiliations:** 1 Denver Health and Hospital Authority, University of Colorado School of Medicine, Denver, Colorado; 2 Denver Health and Hospital Authority, Denver, Colorado; 3 Quidel Laboratories, Kornwestheim, Baden-Wurttemberg, Germany; 4 University of Colorado, School of Medicine, Aurora, CO; 5 Children's Hospital Colorado, Aurora, CO; 6 Denver Health Medical Center, University of Colorado School of Medicine, Denver, Colorado

## Abstract

**Background:**

Among children with acute otitis media (AOM) *S.pneumoniae*, *H.influenzae*, and *M.catarrhalis* are the predominant bacterial otopathogens. There is a high correlation between nasopharyngeal (NP) and middle ear fluid (MEF) organisms during AOM. Thus, NP samples could serve as a surrogate for detection of otopathogens and are more easily collected in a typical practice environment than MEF. Though culture is considered the gold standard for detection, it is time-consuming, which can limit its diagnostic utility to guide clinical care. We aimed to determine the sensitivity, specificity, positive (PPV) and negative predictive value (NPV) for NP qualitative PCR for bacterial otopathogens compared to NP culture.

**Methods:**

Patients age 6-35 months with uncomplicated AOM who were prospectively enrolled in an AOM study in Denver, CO from Jan 2019-Dec 2020 were included. All patients had an NP flocked swab (Eswab^®^, Copan Diagnostics) at enrollment. Otopathogen culture was completed using standard techniques. Nucleic acids were extracted using the NucliSENS® easyMAG® system (Quidel, San Diego, CA) per manufacturer’s instructions. Multiplex RT-PCR for *S.pneumoniae, H.influenzae,* and *M.catarrhalis* was completed using Lyra^®^ (Quidel, San Diego, CA) and AnDiaTec^®^ assay kits (Quidel Germany GmbH, Kornwestheim, Germany). Nucleic acid amplification and detection was completed on the Applied Biosystems^®^ (ABI) 7500 Fast Dx Real-Time PCR Instrument.

**Results:**

Of the 80 children included, 18 (22.5%) had no organism detected on culture, 31 (38.8%) had one and 31 (38.8%) had multiple organisms detected. The most commonly identified organisms on culture were *M.catarrhalis* (42, 52.5%), followed by *S.pneumoniae* (30, 37.5%), and *H.influenzae* (17, 21.3%). Of *H.influenzae* isolates 8 (47.1%) produced beta-lactamase. The sensitivity of PCR was high ( >94%) for all organisms whereas the specificity was lower (50.0-77.8%) and varied by organism (Table). NPV were high ( >96%) for all otopathogens, whereas, PPV ranged from 53.3 to 68.9%. PCR detected 1.6 times more organisms than culture (149 vs. 96).

Sensitivity, specificity, positive and negative predictive value of PCR compared to culture for otopathogens.

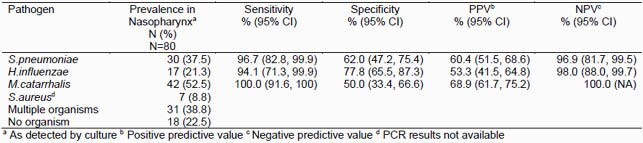

**Conclusion:**

NP PCR has a high predictive value for excluding otopathogens and warrants further exploration as a diagnostic tool to evaluate for otopathogens in children.

**Disclosures:**

**Andreas Bress, PhD**, **Quidel Laboratories- Germany** (Employee) **Richard Egan, PhD**, **Quidel Laboratories** (Employee) **Samuel R. Dominguez, MD, PhD**, **BioFire Diagnostics** (Consultant, Research Grant or Support)**DiaSorin Molecular** (Consultant)**Pfizer** (Grant/Research Support) **Samuel R. Dominguez, MD, PhD**, BioFire (Individual(s) Involved: Self): Consultant, Research Grant or Support; DiaSorin Molecular (Individual(s) Involved: Self): Consultant; Pfizer (Individual(s) Involved: Self): Grant/Research Support

